# The complete mitochondrial genome of *Mesochaetopterus japonicus* (Sedentaria: Chaetopteridae)

**DOI:** 10.1080/23802359.2022.2073840

**Published:** 2022-05-11

**Authors:** Mei Yang, Weina Wang, Xinzheng Li, Jixing Sui

**Affiliations:** a Institute of Oceanology, Chinese Academy of Sciences, Qingdao, People’s Republic of China; b Center for Ocean Mega-Science, Chinese Academy of Sciences, Qingdao, People’s Republic of China; c South China Sea Environmental Monitoring Center, State Oceanic Administration, Guangzhou, People’s Republic of China; d University of Chinese Academy of Sciences, Beijing, People’s Republic of China; e Laboratory for Marine Biology and Biotechnology, Qingdao National Laboratory for Marine Science and Technology, Qingdao, People’s Republic of China

**Keywords:** *Mesochaetopterus japonicus*, mitogenome, Chaetopteridae

## Abstract

The benthic and tube-building polychaete worm *Mesochaetopterus japonicus* is abundantly present on the coast of the western Pacific. Here, we report the complete mitochondrial genome of *M. japonicus*, which is 19,326 bp in length and contains 13 protein-coding genes, 2 rRNA genes and 22 tRNA genes. All 37 genes are encoded on the heavy strand, and AT content is 70.17%. Phylogenetic analyses based on the *M. japonicus* mitogenome combined with previously published polychaete mitogenome data revealed that *M. japonicus* was closely related to *Chaetopterus variopedatus* and *Phyllochaetopterus* sp., all of which belong to Chaetopteridae. The mitochondrial genome of *M. japonicus* could provide useful molecular resources for further research on Polychaeta phylogeny and evolution.

*Mesochaetopterus japonicus* (Fujiwara 1934) is known to be a tube-dwelling polychaete worm and is characterized by three well-defined body regions: anterior, middle and posterior. The species is widely distributed on the coast of the western Pacific (Yang and Sun 1988; Nishi 1999; Nishi and Hsieh 2009) and belongs to the family Chaetopteridae. Chaetopteridae is a globally distributed clade of marine annelids that inhabit areas from intertidal to abyssal depths. There are 76 valid species in four currently recognized genera (Moore et al. 2017): *Chaetopterus* Cuvier, 1830, *Mesochaetopterus* Potts, 1914, *Phyllochaetopterus* Grube, 1863, and *Spiochaetopterus* Sars, 1856 (WoRMS).

The mitochondrial genome (mitogenome) contains abundant genetic information and has a relatively high evolutionary rate (Boore 1999; Barr et al. 2005; Hao et al. 2010), so it is extensively used for studying genetic diversity, phylogeny, molecular evolution, and phylogeography at various taxonomic levels (Gissi et al. 2008; Shen et al. 2017; Lee et al. 2019). However, compared to other polychaetes, information one the Chaetopterid mitogenome is limited, with an examination of the NCBI nr database in August 2021 revealing that only two complete mitogenomes have been published. Herein, we determined the complete mitogenome of the Chaetopterids *M. japonicus* collected from Qingdao (36°03′N, 120°20′E) and deposited in the Marine Biological Museum (Specimen ID: MBM304570, Collection Manger Dr Mei Yang, yangmei@qdio.ac.cn), Institute of Oceanology, Chinese Academy of Sciences, Qingdao, China. Research and collection of animal material was conducted according to the guidelines provided by Science and Technology Ethics Committee of Chinese Academy of Sciences.

Genomic DNA was extracted from the body tissue using the DNeasy Blood and Tissue Kit (Qiagen, Hilden, Germany) and sequenced on the Illumina HiSeq 4000 platform (2 × 150 bp paired-end reads). The mitogenome of *M. japonicus* was *de novo* assembled by using SOAPdenovo 2.04 (Luo et al. 2012) and then annotated by the MITOS2 webserver (Bernt et al. 2013). Finally, the mitogenome was manually corrected.

The complete circular mitogenome of *M. japonicus* is 19,326 bp in length (GenBank Accession no. MZ921947) and contains the typical set of 13 protein-coding genes (PCGs), 22 tRNA genes, 2 rRNA genes, and 1 putative control region. All 37 genes are encoded on the heavy strand, whose nucleotide composition is 34.36% A, 18.70% C, 11.13% G, and 35.81% T, showing a higher content of A + T (70.17%) than G + C (29.83%). The codon ATG was the most popular start codon for *atp6*, *atp8*, *cox2*, *nad3*, *nad4l*, and *nad6*, and the start codon ATT was shared among *cob*, *nad1*, and *nad5*. In particularly, *cox3* and *nad4* begins with the codon ATC, *nad2* begins with the codon ATA, and *cox1* begins with the codon AGA. For stop codon usage, the *cox1* and *nad2* stop with the incomplete stop codon, while the other PCGs terminate with TAA/TAG. Twenty-two tRNA genes varied from 65 to 76 bp in length, and all of them could fold into the typical cloverleaf secondary structure.

The phylogenetic tree was constructed based on 13 PCGs of *M. japonicus* and 21 other polychaetes from three main lineages, including Echiura, Errantia and Sedentaria. The oligochaetes *Acanthobdella peledina* and *Tubifex tubifex* were used as outgroups (Figure 1). Relevant sequences were downloaded from the NCBI database and aligned by MAFFT (Katoh and Standley 2013) with the default parameters. Then phylogenetic relationships were inferred using the maximum-likelihood (ML) method with the GTR + F+R4 model by using IQ-TREE (Nguyen et al. 2015). Bootstrap support was assessed using an ultrafast bootstrap (BP) with 1000 replicates (Hoang et al. 2018). Overall, the relationships between the three lineages (Echiura, Errantia and Sedentaria) followed a well-established annelid phylogeny (Weigert et al. 2016). As the tree indicated, *M. japonicus* was closely related to *Chaetopterus variopedatus* and *Phyllochaetopterus* sp., all of which belong to Chaetopteridae, which is the sister taxon to other Sedentaria taxa.

**Figure 1.**
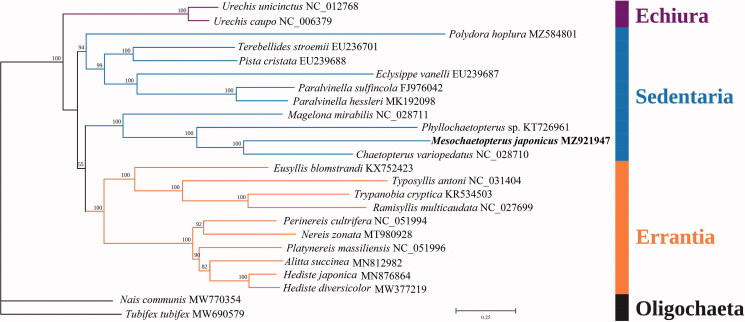
The Maximum-likelihood (ML) phylogenetic tree for *M. japonicus* and the other Polychaeta species based on the concatenated nucleotide sequences of 13 protein-coding genes, and *M. japonicus* is placed with Sedentaria. Bootstrap support values are indicated at each node.

## Data Availability

The genome sequence data that support the findings of this study are openly available in GenBank of NCBI at https://www.ncbi.nlm.nih.gov under the accession no. MZ921947. The associated BioProject, SRA, and Bio-Sample numbers are PRJNA801441, SRR17798510, and SAMN25349843, respectively.
